# Spontaneous CD4^+^ and CD8^+^ T‐cell responses directed against cancer testis antigens are present in the peripheral blood of testicular cancer patients

**DOI:** 10.1002/eji.201646898

**Published:** 2017-06-26

**Authors:** Hayden Pearce, Paul Hutton, Shalini Chaudhri, Emilio Porfiri, Prashant Patel, Richard Viney, Paul Moss

**Affiliations:** ^1^ Institute of Immunology and Immunotherapy College of Medical and Dental Sciences University of Birmingham Birmingham UK; ^2^ University Hospitals NHS Foundation Trust Birmingham UK; ^3^ Institute of Cancer and Genomics College of Medical and Dental Sciences University of Birmingham Birmingham UK

**Keywords:** Cancer testis antigens, MAGE, T cells, Testicular cancer, Tumor immunology

## Abstract

Cancer/testis antigen (CTAg) expression is restricted to spermatogenic cells in an immune‐privileged site within the testis. However, these proteins are expressed aberrantly by malignant cells and T‐cell responses against CTAgs develop in many cancer patients. We investigated the prevalence, magnitude and phenotype of CTAg‐specific T cells in the blood of patients with testicular germ cell tumors (TGCTs). CD8^+^ and CD4^+^ T‐cell responses against MAGE‐A family antigens were present in 44% (20/45) of patients’ samples assayed by ex vivo IFN‐γ ELISPOT. The presence of MAGE‐specific CD8^+^ T cells was further determined following short‐term in vitro expansion through the use of pMHC‐I multimers containing known immunogenic peptides. Longitudinal analysis revealed that the frequency of MAGE‐specific T cells decreased by 89% following orchidectomy suggesting that persistence of tumor antigen is required to sustain CTAg‐specific T‐cell immunity. Notably, this decrease correlated with a decline in the global effector/memory T‐cell pool following treatment. Spontaneous T‐cell immunity against CTAg proteins therefore develops in many patients with testicular cancer and may play an important role in the excellent clinical outcome of patients with this tumor subtype.

## Introduction

Testicular cancer is the most common tumor among young men of 20–34 years of age and is increasing in incidence 1. Testicular germ cell tumors (TGCTs) account for the majority of testicular cancers and consist predominantly of classical seminomas and nonseminomatous germ cell testicular tumors (NSGCTTs). Seminomas are morphologically homogeneous whilst NSGCTTs are more heterogeneous and can be composed of multiple components including embryonic carcinoma, choriocarcinoma cells and teratoma 2. In addition, some testicular germ cell tumors can contain both seminoma and NSGCTT elements and these are classified as mixed germ cell tumors (mGCTs).

All post‐pubertal TGCTs are thought to originate from a pre‐invasive lesion termed germ cell neoplasia in situ (GCNIS) 3. Genome‐wide expression profiling studies suggest that GCNIS cells are derived from pluripotent gonocytes that have survived within the post‐natal testis 4, 5 and subsequent post‐pubertal signals from surrounding somatic cells may initiate the development of an invasive tumor.

Cancer/testis antigens (CTAg) are proteins that are expressed in germ cells but are usually silenced in somatic cells 6. They include the melanoma‐associated antigen (MAGE) family and NY‐ESO‐1 antigen which are attractive candidates for cancer vaccine trials and adoptive cellular immunotherapy 7, 8, 9. Importantly, CTAg expression is observed in many tumor subtypes and incomplete clonal deletion of CTAg reactive thymocytes in the thymus can lead to the development of CTAg‐specific T‐cell responses 10, 11, 12.

Study of CTAg expression in testicular cancer has revealed that MAGE‐A family proteins are expressed frequently in classical seminoma and seminomatous elements of mGCTs but are generally absent in GCNIS 13, 14, 15, 16, 17, 18, 19, 20. Conversely NY‐ESO‐1 expression is limited to pre‐invasive GCNIS 21 and is usually negative in both seminoma and NSGCTT 16, 17, 18 indicating a pattern of downregulation during transition to carcinoma.

As testicular cancer is itself a germ cell tumor we investigated if cellular immunity against CTAg proteins develops in patients with testicular tumors. We find that strong CD8^+^ and CD4^+^ CTAg‐specific T‐cell responses are indeed found in many patients and that the global memory T‐cell pool is also substantially increased at diagnosis. The magnitude of CTAg‐specific responses decreases substantially after treatment and coincides with a reduction in the T‐cell memory pool. These data indicate that natural CTAg‐specific immunity is established in many patients with testicular cancer and comprises a population of short‐lived effector T cells that persist poorly in the absence of antigen.

## Results

### Spontaneous T‐cell responses against MAGE‐A proteins develop in many patients with testicular cancer

Forty‐five patients with a diagnosis of testicular cancer were recruited prior to orchidectomy or within 2 weeks of the operation. PBMCs were stimulated with overlapping peptide pools derived from CTAg proteins prior to analysis using IFN‐γ ELISPOT assay. T‐cell responses were determined against peptide pools representing MAGE‐A1, MAGE‐A3, MAGE‐A4 and NY‐ESO‐1 and were examined in all patients as well as 17 age‐matched male donors.

**Figure 1:**
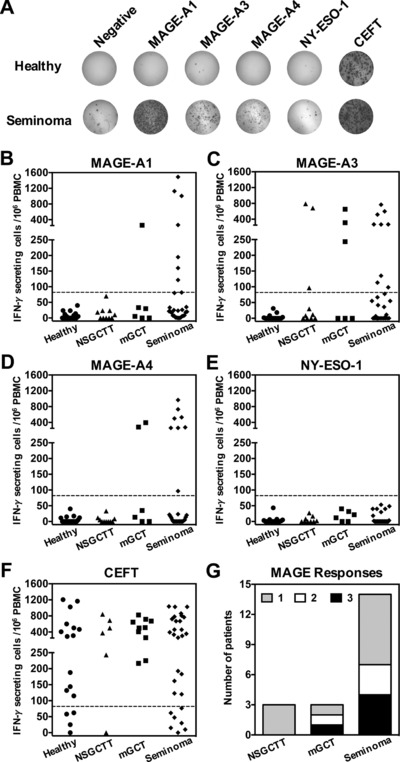
Enumeration of CTAg‐specific T cells in testicular cancer patients and healthy male donors. (A) Representative IFN‐γ ELISPOTs from a healthy male donor and a seminoma patient are shown. PBMCs were stimulated with overlapping peptides spanning the full length of the CTAg protein indicated. Each spot represents a single antigen‐specific IFN‐γ secreting cell. Cells stimulated with DMSO alone served as a negative control and a CEFT peptide pool was used as a positive control. Computational analysis was used to count the spots in each well. (B‐F) Scatter dot plots show the frequency of (B) MAGE‐A1, (C) MAGE‐A3, (D) MAGE‐A4**,** (E) NY‐ESO‐1**,** and **(F)** CEFT**‐**specific T cells in healthy donors (*n* = 17) and patients with NSGCTT (*n* = 10), mGCT (*n* = 6) and seminoma (*n* = 29). Each point represents the mean adjusted value of 2 replicates for each individual patient and is expressed as the frequency of CTAg‐specific T cells /10^6^ PBMCs. Dashed lines represent the threshold for a positive response. (G) An overview of MAGE‐A‐specific responses in TGCT patients by tumor type, showing the number of patients exhibiting a positive response to 1, 2, or all 3 MAGE‐A antigens. Data shown were generated from the indicated number of donor samples for each group.

T‐cell responses against MAGE proteins were observed in many patients with testicular cancer. A representative ELISPOT is shown in Fig. 1A. Specifically, T‐cell responses to MAGE‐A1 were detected in 17% (1/6) and 31% (9/29) of patients with mixed germ cell tumors (mGCT) and seminoma respectively but were absent in patients with non‐seminomatous germ cell testicular tumors (NSGCTT) (Fig. 1B). MAGE‐A3‐specific responses were detected in patients across all testicular tumor types (Fig. 1C) with 31% (9/29) of seminoma, 50% (3/6) of mGCT and 30% (3/10) of NSGCTT patients demonstrating an immune response to this protein. MAGE‐A4 responses were detected in 28% (8/29) of seminoma and 33% (2/6) of mGCT patients (Fig. 1D), but were not identified in patients with NSGCTT. Interestingly, NY‐ESO‐1‐specific T‐cell responses were undetectable in all TGCT patients examined, irrespective of the tumor subtype (Fig. 1E). The majority of patients and healthy donors responded to the CEFT positive control (Fig. 1F). Overall, CTAg‐specific T‐cell responses were detectable in 44% (20/45) of patients prior to adjuvant therapy.

Further analysis revealed that a substantial proportion of patients with seminoma and mGCT exhibited a T‐cell immune response against multiple MAGE‐A family proteins (Fig. 1G). In particular, of those exhibiting at least one response, 50% (7/14) of patients with seminoma and 66% (2/3) of patients with mGCT had responses to at least 2 of the 3 MAGE‐A family antigens that were examined. In contrast, NSGCTT patients generated responses against only the MAGE‐A3 protein. Interestingly, analysis of the immune response in relation to clinical parameters revealed that all NSGCTT patients with a MAGE‐A3 directed immune response had evidence of metastatic disease outside the testis. There was no association between the frequency or magnitude of CTAg‐specific responses and tumor stage in patients with seminoma or mGCT (data not shown).

### MAGE‐specific T cells comprise both CD4^+^ and CD8^+^ T cells and demonstrate a Th1 cytotoxic phenotype

**Figure 2:**
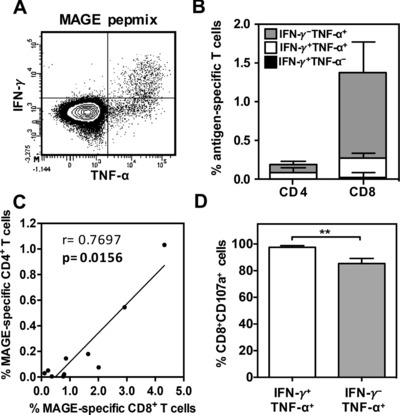
Effector function of MAGE‐A‐specific CD4^+^ and CD8^+^ T cells stimulated with overlapping 15mer peptides. IFN‐γ and TNF‐α production was assessed on both CD4^+^ and CD8^+^ T cells by intracellular cytokine staining following restimulation with overlapping peptides in a cohort of patients demonstrating a positive IFN‐γ ELISPOT (*n* = 10). **(A)** A representative flow cytometry density plot demonstrating the gating strategy used to determine the subset of IFN‐γ^+^TNF‐α^−^, IFN‐γ^−^TNF‐α^+^ and IFN‐γ^+^TNF‐α^+^ T cells following pepmix stimulation is shown. (B) The mean proportion of CD4^+^ and CD8^+^ T cells within each cytokine subset was determined and is represented as a percentage of the total CD4^+^ or CD8^+^ T‐cell pool. Error bars represent SEM. (C) The correlation between the proportion of cytokine‐secreting CD4^+^ T cells and CD8^+^ T cells. Each symbol represents an individual patient and spearman's rank correlation coefficient (r) and significance (p) are reported. **(D)** The proportion of CD107a‐expressing CD8^+^ T cells within subsets of IFN‐γ^+^TNF‐α^+^ and IFN‐γ^−^TNF‐α^+^ cells in response to peptide stimulation. All data excluding (A) are pooled from 10 independent experiments. Data shown as mean + SEM. Data analyzed by Mann–Whitney test, **p < 0.01.

In order to further characterize the CTAg‐specific immune response we next went on to define the T‐cell subtype and effector function in a group of patients with an established MAGE‐specific response (*n* = 10). PBMCs were stimulated with overlapping peptides in short‐term cultures and then intracellular cytokine staining was used to examine IFN‐γ and TNF‐α production (Fig. 2A). As expected given their previous identification through IFN‐γ ELISPOT, all patients demonstrated intracellular IFN‐γ production following peptide stimulation (data not shown). However, it was noteworthy that all patients also exhibited a high frequency of MAGE‐specific cells which produced TNF‐α but not IFN‐γ and this IFN‐γ^−^TNF‐α^+^ phenotype was more pronounced within the CD8^+^ T‐cell compartment (Fig. 2B). The frequency of MAGE‐specific CD8^+^ T cells was 6‐fold greater than MAGE‐specific CD4^+^ T cells, yet a strong positive relationship within individual donors was observed (r = 0.7697, *p* = 0.0156) (Fig. 2C) suggesting the development of a coordinated CD4^+^ and CD8^+^ T‐cell response against such antigens.

Finally, we determined the cytotoxic capacity of CD8^+^ T cells using CD107a surface mobilization as a marker of degranulation, and analyzed this in relation to the cytokine secretion profile. Importantly, although the majority of IFN‐γ^+^TNF‐α^+^ and IFN‐γ^−^TNF‐α^+^ CD8^+^ T cells demonstrated surface mobilization of CD107a in response to peptide stimulation (mean: 97.5% vs. 85.3%) (Fig. 2D), nearly 15% of the latter subset failed to undergo degranulation (*p* = 0.0054).

### MAGE‐specific CD8^+^ T cells can be identified through staining with immunodominant pMHC‐I multimers

**Figure 3:**
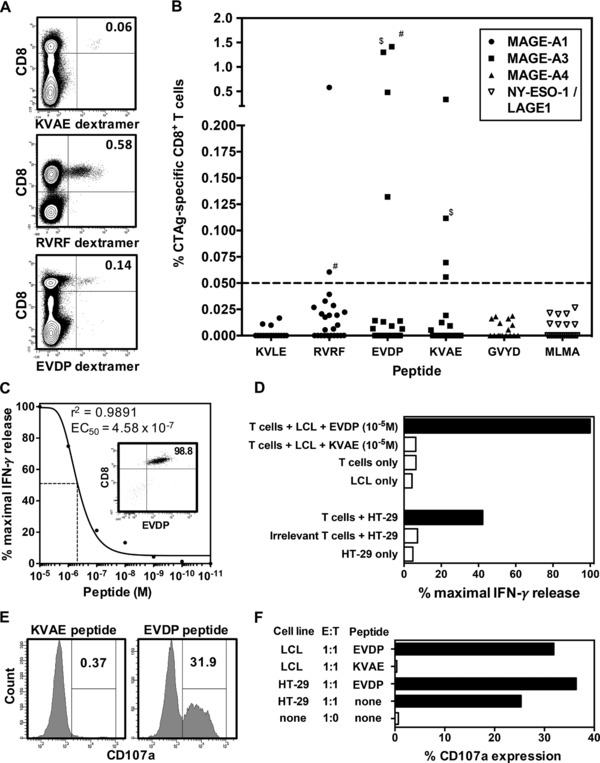
Detection and functional characterization of CD8^+^ T cells specific to immunodominant MAGE‐A family epitopes in TGCT patients. PBMCs from patients were stimulated with appropriate CTAg peptide in vitro for 10 days followed by staining with matched pMHC‐I dextramer. (A) Representative flow cytometry contour plots of KVAE (top), RVRF, (middle) and EVDP (bottom)–specific CD8^+^ T cells in TGCT patients prior to chemotherapy are shown. (B) The frequency of CTAg‐specific T cells represented as a percentage of the total CD8^+^ T‐cell pool (*n* = 22). The CTAg peptides within each pMHC‐I dextramer are represented on the x‐axis. The dashed line represents the threshold for a positive response. # represents points from the same patient with a simultaneous MAGE‐A1 and MAGE‐A3 response. $ represents points from the same patient who displayed responses to two different MAGE‐A3 epitopes. (C) A MAGE‐A3_EVDP_ ‐specific CD8^+^ T‐cell clone was generated from a seminoma patient (inset), and its avidity for EVDP peptide was determined by peptide titration analysis. The avidity of the TCR was defined as the peptide concentration required to induce half maximal IFN‐γ release in an ELISA assay (dashed line: EC_50_). Data shown are of a single T‐cell clone assayed in triplicate. (D) EVDP‐specific T cells were co‐cultured with target cells and response was measured using IFN‐γ production by ELISA. EVDP‐specific T cells were co‐cultured with EVDP loaded LCLs or MAGE‐A3 expressing HT‐29 cell line. Spontaneous IFN‐γ production (open bars) was assessed by culture of T cells alone, LCL alone, and HT‐29 alone. Data shown are the mean IFN‐γ release of a single T‐cell clone assayed in triplicate. LCL pulsed with irrelevant peptide (KVAE) was also used to verify specificity of the clone. (E) The cytotoxic potential of a single EVDP‐specific T‐cell clone was assessed in a degranulation assay using CD107a mobilization. Flow cytometry histograms show the proportion of T cells expressing CD107a following stimulation with LCL pulsed with irrelevant peptide (KVAE) and test peptide (EVDP). (F) The induction of surface CD107a expression was determined following co‐culture with EVDP pulsed LCL and HT‐29 cell line at an E:T ratio of 1:1, along with appropriate controls. CD107a expression was represented as a percentage of total EVDP‐specific T cells.

Following identification of MAGE‐specific CD8^+^ T cells through cytokine production we next investigated if CTAg‐specific T cells could be identified through the use of staining with HLA‐peptide dextramers containing immunodominant peptides from MAGE‐A1, MAGE‐A3, MAGE‐A4 and/or NY‐ESO‐1/LAGE‐1 (Table 1). Freshly isolated PBMCs were expanded with peptide for 10 days prior to dextramer staining (Fig. 3A) and a positive response was reported when the frequency of MAGE‐specific T cells was greater than 0.05% of the total CD8^+^ T‐cell pool (1 in 2000). MAGE‐specific responses were detected in 36% (8/22) of patients (Fig. 3B). MAGE‐A1 (RVRF)‐specific T‐cell responses were detected in 9% (2/22) of patients and almost a third (32%) of patients had detectable MAGE‐A3‐specific T cells (EVDP and/or KVAE). Importantly, whereas both patients who demonstrated MAGE‐A1 (RVRF)‐specific responses had a diagnosis of seminoma, MAGE‐A3 responses (EVDP and KVAE) were detected in patients across all tumor subtypes (data not shown). MAGE‐A4 (GVYD) and NY‐ESO‐1/LAGE1 (MLMA)‐specific T‐cell responses were not detected in this patient cohort. These results confirm that CD8^+^ T‐cell responses against a range of MAGE proteins are present in patients with testicular cancer and that these can be identified within the peripheral circulation through the use of pMHC‐I multimers.

**Table 1:** Immunodominant peptides used in dextramer assays

Protein	HLA restriction	HLA frequency (%)	Peptide sequence	Position
MAGE‐A1	A2	42.6	KVLEYVIKV	278‐286
	B7	21.4	RVRFFFPSL	289‐298
MAGE‐A3	A1	29.1	EVDPIGHLY	168‐176
	A2	42.6	KVAELVHFL	112‐120
MAGE‐A4	A2	42.6	GVYDGREHTV	230‐239
NY‐ESO‐1/LAGE‐1	A2	42.6	MLMAQEALAFL	1 ‐11 (ORF2)

### MAGE‐specific CD8^+^ T‐cell clones display cytotoxic activity against tumor cells

In order to investigate the avidity and function of CTAg‐specific T cells in patients with testicular cancer we next went on to generate a primary CTAg‐specific T‐cell clone from a patient with seminoma. A CD8^+^ T‐cell clone specific for the EVDP peptide derived from MAGE‐A3 and restricted by HLA‐A1 was isolated from peripheral blood taken at disease presentation (Fig. 3C, inset). Interestingly, the clone exhibited a relatively low avidity for peptide with 50% maximal IFN‐γ release (EC_50_) at a peptide concentration of 4.58 × 10^−7^ M (Fig. 3C). Despite this the CD8^+^ clone demonstrated strong and specific recognition of both peptide‐pulsed LCLs and the HT‐29 cell line, indicating the ability to recognize tumor cells which express endogenous CTAg protein (Fig. 3D). Finally, the cytotoxic potential of the clone was examined through the use of the CD107a mobilization assay. T cells were co‐cultured with either EVDP peptide‐loaded LCL or HT‐29 cells and expression of CD107a was then examined on effector cells. CD107a expression was observed in both cases, indicating both that the T‐cell clone has cytotoxic capacity and that this can be induced following endogenous antigen presentation of the MAGE protein (Fig. 3E and F).

### The frequency and magnitude of the CTAg‐specific T‐cell response decrease markedly after treatment

**Figure 4:**
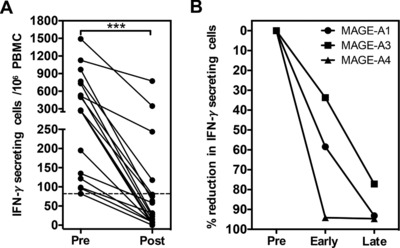
Longitudinal analysis of the global CTAg‐specific T‐cell response in patients with testicular cancer. (A) The frequency of MAGE‐A‐specific T cells was measured by IFN‐γ ELISPOT before (Pre) and after the completion of adjuvant therapy (Post; ≥6 months) for a cohort of seminoma patients in whom a positive response to MAGE‐A1, ‐A3, and/or ‐A4 had been detected prior to treatment, and where a post‐treatment sample was available. A total of 18 MAGE‐A responses from 10 patients were assayed in duplicate. (B) The percent reduction of MAGE‐specific T cells at two timepoints (Early, <3 months; Late, ≥6 months) after treatment, relative to the pre‐treatment (Pre) frequency. Data analyzed by Wilcoxon matched‐pairs signed‐rank test, ***p < 0.001.

We next went on to investigate the profile of CTAg‐specific immune responses during and after chemotherapy. Blood samples were obtained at several time points from a cohort of seminoma patients in whom we had detected a positive CTAg‐specific T‐cell response prior to treatment. The frequency of MAGE‐specific T cells was assessed at least 3 months after completion of initial treatment and compared to that prior to adjuvant therapy (Fig. 4A). Interestingly, the MAGE‐specific immune response remained detectable in only 22% of cases (4/18) at the later timepoint.

We next investigated the temporal kinetics of CTAg‐specific immunity to individual MAGE proteins within the observed period of decline during follow up. Interestingly, a progressive decrease in the frequency of antigen‐specific T cells against each MAGE protein was observed (Fig. 4B). Specifically, the magnitude of the MAGE‐specific T‐cell response fell by 89% overall during follow‐up compared to pre‐treatment values (median: 29.6 vs. 273, *p* = 0.0002). Importantly, we also studied if MAGE‐specific T‐cell responses were induced following adjuvant treatment in any patients in whom they had not been present at diagnosis but no such responses were observed (data not shown) suggesting that chemotherapy does not induce CTAg‐directed anti‐tumor immunity.

### Testicular cancer patients have a marked expansion in the T‐cell memory pool at disease presentation

**Figure 5:**
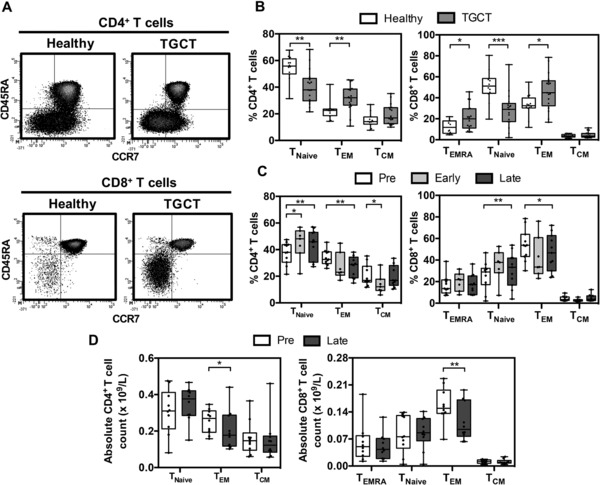
The association between the decline of CTAg‐specific immunity and the global effector memory (T_EM_) T‐cell pool. **(A)** Representative flow cytometry density plots of CCR7 and CD45RA expression on CD4^+^ and CD8^+^ T cells used to define T_EMRA_, T_Naive_, T_EM_ and T_CM_ memory subsets in healthy donors and TGCT patients. (B) The proportion of memory CD4^+^ (left) and CD8^+^ (right) T‐cell subsets in TGCT patients prior to post‐orchidectomy therapy (*n* = 17) compared with that in healthy donors (*n* = 12) as measured in (A) are shown. Data are shown as median + interquartile range (box) and min. and max. values (whiskers) of *n* = 17 (TGCT) and *n* = 12 (healthy) samples. Symbols represent individual donors. Significance evaluated by Mann–Whitney test. (C, D) The proportion (C) and absolute numbers (D) of CD4^+^ and CD8^+^ T cells within defined memory subsets before therapy (Pre, *n* = 11) and at Early (<3 months, *n* = 7) and/or Late (≥6 months, *n* = 11) timepoints post treatment were also measured. Data are shown as median + interquartile range (box) and min. and max. values (whiskers) of the indicated number of donor samples. Data analyzed by Wilcoxon matched‐pairs signed‐rank test, *p < 0.05, **p < 0.01, ***p < 0.001.

In order to explore potential mechanisms that might underlie the marked attrition of MAGE‐specific T cells after treatment we went on to investigate the composition of the peripheral T‐cell memory pool in patients both at diagnosis and following treatment. These values were then compared to the proportions of memory cells in age and gender matched healthy controls. The pattern of CD45RA and CCR7 expression was used to define the proportion of naive (CD45RA^+^CCR7^+^, T_Naive_), central memory (CD45RA^−^CCR7^+^, T_CM_), CD45RA^−^ effector/memory (CD45RA^−^CCR7^−^, T_EM_) and CD45RA^+^ revertant effector (CD45RA^+^CCR7^−^, T_EMRA_) cells within the CD4^+^ and CD8^+^ populations of healthy donors and TGCT patients (Fig. 5A).

Interestingly, patients with testicular cancer were shown to have a markedly increased proportion of T cells within the memory pool and a significant reduction in the proportion of naïve cells. Specifically, within healthy donors the proportion of CD45RA^+^CCR7^+^ T_Naive_ cells represented 56% and 51% within the CD4^+^ and CD8^+^ repertoires respectively. These values were reduced by 25–40% to levels of 38 and 30% within the patient group (*p* = 0.003 and *p* = 0.0008, respectively) (Fig. 5B). In contrast, the proportion of CD4^+^ T_EM_ cells was increased from 23% within healthy donors to 32% within TGCT patients (*p* = 0.0028). CD8^+^ T_EM_ cells were also increased by around 35% from values of 32% within healthy donors to 45% within the patient group (*p* = 0.0284). Furthermore, the CD8^+^ T_EMRA_ population was also increased in the patient group compared to the healthy cohort (*p* = 0.0354). These data suggest that tumor development is associated with the generation of large numbers of antigen‐experienced T cells that inflate the peripheral blood memory T‐cell pool.

### Treatment of testicular cancer leads to a reduction in the peripheral blood memory T‐cell pool

Given our observation that patients with TGCT exhibited an average 30% increase in the size of the CD4^+^ and CD8^+^ T‐cell memory pool at the time of presentation, we next went on to assess the effect of treatment on the proportion of naïve and memory T cells. Interestingly, prospective analysis revealed that the proportion of T cells within the effector/memory T‐cell pool gradually declined after treatment whereas the naïve T‐cell pool increased (Fig. 5C). In particular, the naïve CD4^+^ and CD8^+^ subsets increased significantly from 38 and 29% to values of 46 and 34% respectively at ≥6 months after treatment. Reciprocally, the CD4^+^ and CD8^+^ effector memory (T_EM_) pools fell from 33 and 54% to values of 28 and 46% respectively. Furthermore, we investigated the absolute numbers of memory T‐cell subsets before and after treatment and found a substantial reduction in both the CD4^+^ (*p* = 0.0488) and CD8^+^ (*p* = 0.0059) T_EM_ populations (Fig. 5D). Importantly, there was no significant change in the number of T_Naive_ cells or the other memory subsets suggesting an overall loss of T_EM_ cells from the T‐cell pool during follow‐up. These data indicate that the inflation within the memory T‐cell pool that was observed at the time of diagnosis is comprised largely of short‐lived effector cells that are eroded relatively rapidly following treatment.

## Discussion

Burnet's original model of cancer immune surveillance 22 has now been developed and extended to include the phases of tumor elimination, equilibrium and escape 23. Immune‐mediated elimination of transformed cells requires the recognition of cancer‐specific epitopes and cancer testis antigens (CTAgs) are one such class of potential target. Cellular and humoral CTAg‐specific immune responses have been observed in many tumor subtypes and are suggested to play an important role in the control of disease progression 24. In this study, we frequently observed spontaneous CTAg‐specific T‐cell responses ex vivo (by IFN‐γ ELISPOT) in patients with testicular cancer but not in healthy male individuals. In addition, CD8^+^ T cells specific to known immunogenic epitopes of MAGE‐A family proteins were detectable in testicular cancer patients (by pMHC‐I multimer staining) following short‐term in vitro peptide stimulation cultures.

It is interesting to speculate on the mechanisms that underlie the priming of immune responses against CTAg proteins in patients with testicular cancer. CTAg expression has been reported in testicular germ cell tumors and appears to be largely restricted to tumors containing seminomatous elements. Indeed, classical seminomas express several MAGE‐A family proteins but expression is absent or rare in NSGCTTs 14, 15, 17, 19, 20, 25. Interestingly, this pattern reflects the profile of CTAg‐specific immunity that was observed in the patient cohort, with T‐cell immunity being detected frequently in patients with seminoma and mixed germ cell tumors. One intriguing observation was that patients with seminoma and mGCT often developed T‐cell responses against several MAGE‐A family proteins whereas patients with NSGCTT only exhibited immune responses with specificity for MAGE‐A3. Furthermore, these responses to MAGE‐A3 were seen only in patients with NSGCTT who had metastatic disease outside the testis. MAGE‐A3 has been described as a mediator of extracellular matrix protein function which promotes tumor cell migration 26 and it is therefore possible that expression of MAGE‐A3 is activated during NSGCTT metastasis and that a specific immune response is subsequently induced at the secondary tumor site. In addition, it is unlikely that immunogenic CTAg protein was derived from normal testicular tissue since NY‐ESO‐1‐specific immune responses were not observed in the present study even though NY‐ESO‐1 is expressed strongly by normal testicular spermatogenic cells 21. The lack of NY‐ESO‐1‐specific T‐cell responses in our patients could be explained by potential down regulation of NY‐ESO‐1 protein during the malignant transformation from pre‐malignant GCNIS to clinically overt disease 21.

Cross‐presentation of tumor antigens by dendritic cells at the tumor site or within tumor‐draining lymph nodes is generally considered the primary mode for priming naive tumor‐specific CD8^+^ T cells 27, 28. Effective antigen presentation combined with the high level of CTAg protein that is available in patients with testicular cancer is likely to lead to the profound level of T‐cell expansion that is observed in this setting. In this regard, it is noteworthy that a large immune infiltrate is seen in virtually every case of seminoma 20, which is likely to allow substantial exposure of tumor antigens to the immune system.

We observed that a large proportion of MAGE‐specific CD8^+^ T cells secreted TNF‐α alone following stimulation with CTAg peptides and that this subset was marginally less cytotoxic than cells which also produced IFN‐γ. Previous studies in hepatocellular carcinoma 29, 30 and breast cancer 10 patients have shown a similar phenomenon whereby tumor antigen‐specific CD8^+^ T cells demonstrated a lack of IFN‐γ production. As such, tumor‐specific CD8^+^ T‐cell responses may exhibit unique patterns of functional competence and at a practical level this indicates that the choice of IFN‐γ ELIPSOT as a functional test is likely to have underestimated the true frequency of CTAg‐specific T cells.

A further interesting aspect of MAGE‐specific T‐cell immunity was the detection of both cytotoxic CD8^+^ and CD4^+^ T‐cell immunity in many patients. Antigen‐specific CD4^+^ T cells are required for the induction of cross‐primed CD8^+^ T cells responses 31 and also support the functional activity of secondary immune responses 32, 33, 34. These data suggest that immunotherapeutic approaches which seek to induce or expand CTAg‐specific CD8^+^ T‐cell immunity should include appropriate epitopes for CD4^+^ T cells.

Longitudinal analysis in TGCT patients demonstrated a dramatic reduction in the number of MAGE‐specific T cells within the blood following treatment. The initial blood samples had been taken shortly after tumor removal but before adjuvant chemotherapy. It is possible that adjuvant chemotherapy may trigger apoptosis of proliferating tumor‐reactive immune cells, including the loss of MAGE‐specific T cells. Alternatively, the decline of CTAg‐specific immunity is likely to reflect the absence of available CTAg antigen following orchidectomy and as such is similar to the kinetics of decline of adaptive immunity following the clearance of acute viral and bacterial antigens 35, 36. In support of this, a recent publication showed that placental‐specific T‐cell immune responses can be established during pregnancy but that these are short‐lived and became undetectable in the periphery after delivery 37. It also remains possible that CTAg‐specific immunity does in fact persist but becomes unresponsive to antigenic stimulation due to increased expression of inhibitory checkpoint receptors such as PD‐1 on the cell surface, or an increase in regulatory cells such as T‐regulatory cells (Tregs) or myeloid‐derived suppressor cells (MDSCs) in the blood following treatment, as has been described previously for HPV‐specific responses in oropharyngeal patients 38. Intriguingly, the fall in CTAg‐specific immunity correlated with the concurrent decline in memory T‐cell subsets and so it is tempting to speculate that MAGE‐A‐specific T cells represent a component of a potentially sizeable tumor‐specific effector T‐cell repertoire in patients at the time of diagnosis.

Patients with testicular cancer have an excellent clinical outcome with a cure rate of over 95%. In addition, patients with seminoma respond very well to therapy even in the setting of metastatic disease. It is tempting to consider the potential role of tumor‐specific immune responses within this response as the efficacy of chemotherapy may be partially mediated through immunological mechanisms 39. The expression of HLA proteins on testicular tumors is low which raises important questions regarding the potential mechanisms by which a tumor‐specific T‐cell response may have some benefit in this disease 40, 41. The lack of surface HLA expression on tumor cells would mitigate against direct tumor cell lysis but recognition of local tissue could lead to inflammatory responses that may be capable of mediating non‐specific activity against tumor tissue.

Unfortunately, due to the limited availability of fresh autologous tumor tissue, we were unable to correlate expression of MAGE antigens with the presence of peripheral MAGE‐specific T‐cell populations, or determine if MAGE‐specific T cells were present at the tumor site. However, a previous study demonstrated the presence of MAGE‐A3‐specific CD8^+^ T cells amongst TIL in a patient with seminoma 25, indicating that CTAg‐specific T cells can infiltrate tumor tissue.

In summary, our data demonstrates that MAGE‐specific T‐cell responses frequently develop in many patients with testicular cancer, yet these progressively decline as the disease comes under control. As such patients with testicular cancer represent an excellent opportunity to investigate novel aspects of tumor‐specific immune responses. Moreover, it is possible that strong tumor‐specific immunity plays an important role in mediating the excellent clinical outcome for patients with this condition.

## Materials and methods

### Study participants

Venous blood samples were obtained from patients prior to, and after completion of, post‐orchidectomy therapy for the treatment of testicular cancer at the New Queen Elizabeth Hospital, Birmingham, UK (*n* = 72). All patients had recently undergone radical orchidectomy to remove the involved testicle. Up to 30 mL heparinized blood was obtained from patients prior to chemotherapy and at regular intervals following treatment, which coincided with clinic visits. Up to 50 mL of blood was donated by healthy male volunteers (*n* = 22), which were used as controls. Patient and healthy donor characteristics are shown is Supporting Information Table 1. Written informed patient consent and local ethical committee approval (South Birmingham research ethics committee LREC reference 09/H1207/161, study reference RRK3953) were obtained prior to sample collection. Patients were 18 years or above and competent to give full informed consent.

### Isolation of peripheral blood mononuclear cells (PBMCs)

PBMCs were isolated from heparinized blood by density gradient centrifugation over Lymphoprep (Axis‐Shield) within 4 h of collection. PBMCs were either assayed fresh (for dextramer analysis) or cryopreserved in liquid nitrogen (for memory phenotyping and ELISPOT analysis) in media containing 90% FCS and 10% DMSO.

### Memory phenotyping of T cells

PBMCs (5 × 10^5^) were resuspended in 100 μL MACS buffer (PBS, 0.5% BSA, 2 mM EDTA), and surface stained with fluorochrome‐conjugated antibodies on ice for 30 min to identify memory T‐cell subsets (CD3‐APCCy7, CD4‐PerCPCy5.5, CD8‐AmCyan, CCR7‐PE and CD45RA‐efluor450). Propidium Iodide (PI) was added prior to cytometric analysis to exclude non‐viable cells. Acquisition was carried out with an LSRII flow cytometer (BD Biosciences) using FACSDiva software.

### IFN‐γ ELISPOT Assay

T‐cell responses to CTAgs were measured for each individual patient in duplicate across all available timepoints using IFN‐γ ELISPOT assays conducted against pools of overlapping 15mer peptides (pepmixes; JPT Peptide Technologies) spanning the entire amino acid sequence of each antigen. Previously frozen PBMCs (5 × 10^6^) from TGCT patients and healthy male donors were seeded in a 24‐well cell culture plate and rested overnight at 37°C to allow cells to recover from cryopreservation. A Multiscreen 96‐well plate (Millipore) was coated with IFN‐γ capture antibody (Mabtech) at 4°C overnight. PBMCs were harvested the following day for ELISPOT assay setup and seeded at 2.5–3.5 × 10^5^ cells/well. For background spot determination, a negative control containing DMSO solvent only was used for each patient sample. Test condition wells were incubated with MAGE‐A1, MAGE‐A3, MAGE‐A4 or NY‐ESO‐1 pepmixes (1 μg of each peptide/mL). A CEFT peptide mix (immunogenic peptides derived from CMV, EBV, Flu and tetanus antigens) was also included as a positive control. The ELISPOT plates were incubated for 16–18 h at 37°C and developed as per manufacturer's instructions (Mabtech). IFN‐γ spots were counted using an AID automated ELISPOT reader. Each spot is representative of a single reactive IFN‐γ secreting T cell. Mean spot counts for negative control wells were subtracted from those for the test wells to determine the frequency of antigen specific T cells. Positive CTAg responses were defined as the recognition of pepmixes for which the mean adjusted counts were ≥2‐fold higher than that of the highest adjusted spot count observed in our healthy male control cohort.

### Intracellular cytokine staining and CD107a mobilization assay following antigen restimulation

Flow cytometry was used to determine whether responses were attributable to antigen‐specific CD4^+^ and/or CD8^+^ T cells. Production of effector cytokines IFN‐γ and TNF‐α, and CD107a mobilization was simultaneously examined following restimulation with pepmixes. CD107a mobilization to the surface of responding T cells is a prerequisite for degranulation and was used here as a marker of cytotoxic potential of CD8^+^ T cells. Briefly, previously cryopreserved PBMCs of patients (*n* = 10) with known CTAg responses by ELISPOT were resuspended in RPMI 1640 media containing 10% FCS at a concentration of 2 × 10^6^ cells/mL. Cell were stimulated with pepmixes overnight, then cultured for 10 days in the presence of 50 U/mL IL‐2 from day 3. Cells were then restimulated with pepmixes in the presence of 20 ng/mL CD107a‐FITC antibody. After 1 h, protein transport inhibitors (5.3 mM brefeldin‐A, 1 mM monensin) were added to each well. Cells were incubated for a further 4 h, and then surface stained with CD3‐APC‐Cy7, CD4‐PerCP‐Cy5.5 and CD8‐AmCyan. Cells were washed in PBS, then fixed and permeabilised with 1% PFA and 0.5% saponin, respectively. Cells were stained intracellularly for the cytokines IFN‐γ‐AF700 and TNF‐α‐PECy7, and then analyzed by flow cytometry. Unstimulated cells (DMSO only) and CEFT peptide pool served as negative and positive controls, respectively.

### Expansion of antigen‐experienced CTAg‐specific CD8^+^ T cells in short‐term T‐cell line cultures

Fresh PBMCs were incubated with 10 μg/mL of each peptide (Table 1) in serum‐free RPMI 1640 media for 1 h with gently agitation. Cells were washed and resuspended in RPMI 1640 media supplemented with 10% human serum, IL‐7 (25 ng/mL), and IL‐15 (5 ng/mL) at a concentration of 2 × 10^6^ cells/mL. IL‐2 (50 U/mL) was added to the cultures from day 3. Cells were cultured for between 10 and 12 days to allow for sufficient T‐cell expansion. Dextramer (pMHC‐I multimer; Immudex) staining was used to identify antigen‐specific CD8^+^ T cells following short‐term culture.

### Identification of CTAg‐specific CD8^+^ T cells using pMHC‐I dextramers

T‐cell line cultures were stained with pMHC‐I dextramers for 20 min at RT in MACS buffer, and washed once prior to surface staining. Cells were surface stained with CD3‐APC‐Cy7, CD4‐FITC and CD8‐PC5 on ice for 30 min, then washed twice before cytometric analysis. PI was added just prior to cytometric analysis to exclude non‐viable cells. Cells were first gated for lymphocytes (SSC‐A vs. FSC‐A), followed by gating for viable CD8^+^ T cells (PI^−^CD3^+^CD4^−^CD8^+^). Dual CD8 and pMHC‐I dextramer expression was then determined from this gated population. A positive response was reported when the frequency of antigen‐specific dextramer‐stained CD8^+^ T cells was ≥0.05% of total CD8^+^ T cells, a threshold described in a number of previous studies 42, 43.

### Generation of MAGE‐A3‐specific T‐cell clones

MAGE‐A3_EVDP_ dextramer positive cells were enriched using anti‐PE beads and a double MS column isolation procedure under sterile conditions, following the manufacturer's instructions (Miltenyi Biotec). The isolated cells were then cloned by limiting dilution over irradiated (40 Gy) PHA‐stimulated allogeneic PBMCs and peptide‐loaded partially‐matched lymphoblastic cell lines (LCL) in RPMI 1640 supplemented with 5% human serum, 5% FCS, IL‐2 (100 U/mL), IL‐15 (5 ng/mL), and IL‐21 (2 ng/mL). For the maintenance of the T‐cell clones, media containing IL‐2 (100 U/mL) and IL‐15 (5 ng/mL) was added twice weekly.

### Functional assessment of an EVDP‐specific T‐cell clone

Potential MAGE‐A3_EVDP_‐specific T‐cell clones were initially screened by staining with dextramer and a single positive clone was studied further. Effector function was measured by the release of IFN‐γ into culture supernatant following 16 h incubation with targets cells. Target cells were LCL loaded with relevant peptide (EVDP), LCL loaded with irrelevant peptide (KVAE) and a cell line expressing HLA‐A1 and MAGE‐A3 (HT‐29) 44, 45. Assay controls included T‐cell clone only, LCL only and HT‐29 only. The culture supernatant medium was harvested and assayed for IFN‐γ by ELISA (Mabtech) in accordance with the manufacturer's instructions. For TCR avidity assays, peptide was titrated from 10^−5^ to 10^−11^ M and expressed as a percentage of maximal IFN‐γ release. CD107a mobilization assay was performed as described above. T cells were stimulated with LCL loaded with either 10 μg/mL EVDP or KVAE peptides, or with the HT‐29 cell line ± EVDP peptide.

## Data handling and statistical analysis

Statistical analysis was performed using GraphPad Prism version 5 (GraphPad Software). To determine differences between two independent groups, a non‐parametric Mann–Whitney test was performed. A Wilcoxon matched‐pairs signed‐rank test was used to compare non‐parametric paired data. A linear regression was performed to assess the relationship between two variables. Spearman's nonparametric test was used to determine correlations. Normal distribution was assessed using the D'Agostino & Pearson test, where appropriate. A *p* value <0.05 was considered statistically significant.

## Conflict of interest

The authors declare no commercial or financial conflict of interest.

**Abbreviations**
TGCT: testicular germ cell tumor
NSGCTT: non‐seminomatous germ cell testicular tumor
mGCT: mixed germ cell tumor
MAGE: melanoma‐associated antigen
LCLs: lymphoblastic cell lines
PBMCs: peripheral blood mononuclear cells
pMHC‐I: peptide major histocompatibility antigen class‐I

## Supporting information

Supporting Information Table 1 Patient characteristics

Peer review correspondence
