# Supplementary material for: Spontaneous CD4+ and CD8+ T‐cell responses directed against cancer testis antigens are present in the peripheral blood of testicular cancer patients
Source: Eur J Immunol. 2017 Jun 26;47(7):1232–42. doi: 10.1002/eji.201646898 (PMC5519936; doi:10.1002/eji.201646898)
Supplement: Supplementary file 1 — Supporting Information Table 1 Patient characteristics [file EJI-47-1232-s001.pdf]

European Journal of Immunology

Supporting Information
for
DOI 10.1002/eji.201646898

Hayden Pearce, Paul Hutton, Shalini Chaudhri, Emilio Porfiri, Prashant Patel, Richard Viney and Paul Moss

Spontaneous $CD4^+$ and $CD8^+$ T-cell responses directed against cancer testis antigens are present in the peripheral blood of testicular cancer patients

© 2017 The Authors. *European Journal of Immunology* published by WILEY-VCH Verlag GmbH & Co. KGaA, Weinheim.

Supplementary Table. 1 Patient characteristics

		Frequency (number)
**Tumour subtype**	All subtypes	100% (72)
	Seminoma	66% (48)
	NSGCTT	20% (14)
	mGCT	14% (10)
**Tumour stage**		
Seminoma	Stage 1	81% (39)
	Stage 2a	6% (3)
	Stage 2b	11% (5)
	Stage 3	2% (1)
NSGCTT	Stage 1 (localised)	36% (5)
	LVI -	40% (2)
	LVI +	60% (3)
	Stage 2 (metastatic)	64% (9)
	IGCCCG[a] - Good	89% (8)
	IGCCCG - Intermediate	11% (1)
mGCT	Stage 1 (localised)	90% (9)
	LVI −	67% (6)
	LVI +	33% (3)
	Stage 2 (metastatic)	10% (1)
	IGCCCG - Good	100% (1)
**Treatment**		
Seminoma	1 x Carboplatin AUC7	81% (39)
	3/4 x EP120	17% (8)
	Radiotherapy	2% (1)
NSGCTT	1 x BEP165	21% (3)
	2 x BEP120	21% (3)
	3/4 x BEP165	58% (8)
mGCT	1 x BEP165	20% (2)
	2 x BEP120	60% (6)
	3 x BEP165	10% (1)
	Surveillance	10% (1)
**Recurrence within 2 years**		3% (2/72) patients
**Age**		Median years (Range)
All TGCT patients		37 (20 - 57)
Seminoma		41 (21 - 57)
NSGCTT		25 (20 - 36)
mGCT		40 (30 - 48)
Healthy donors		30 (23 -51)

Abbreviations: LVI, Lymphovascular invasion; IGCCCG, International Germ Cell Cancer Collaborative Group; BEP, Bleomycin- Etoposide-Cisplatin; EP Etoposide-Cisplatin

[a]Prognostic classification of metastatic germ cell tumours
