# Supplementary material for: Spontaneous CD4+ and CD8+ T‐cell responses directed against cancer testis antigens are present in the peripheral blood of testicular cancer patients
Source: Eur J Immunol. 2017 Jun 26;47(7):1232–42. doi: 10.1002/eji.201646898 (PMC5519936; doi:10.1002/eji.201646898)
Supplement: Supplementary file 2 — Peer review correspondence [file EJI-47-1232-s002.pdf]

Peer review correspondence

European Journal of Immunology

Spontaneous CD4$^+$ and CD8$^+$ T-cell responses directed against cancer testis antigens are present in the peripheral blood of testicular cancer patients

Hayden Pearce, Paul Hutton, Shalini Chaudhri, Emilio Porfiri, Prashant Patel, Richard Viney and Paul Moss

Corresponding author: Paul Moss, Institute of Immunology and Immunotherapy, College of Medical and Dental Sciences, University of Birmingham, Birmingham, United Kingdom

---

Review Timeline:	Submission date:	20 December 2016
	First editorial decision:	27 January 2017
	Revision received:	17 March 2017
	Accepted:	22 May 2017

Handling Executive Committee member: Prof. Iain McInnes

Please note that the correspondence below does not include the standard editorial instructions regarding preparation and submission of revised manuscripts, only the scientific revisions requested and addressed.

First Editorial Decision – 27 January 2017

Dear Dr. Pearce,

We are sorry for a delay in the peer review of your manuscript ID eji.201646898 entitled "Spontaneous CD4+ and CD8+ T cell responses directed against cancer testis antigens are present in the peripheral blood of testicular cancer patients" which you submitted to the European Journal of Immunology. There was a delay in receiving on of the reports. The comments of the referees are included at the bottom of this letter.

A revised version of your manuscript that takes into account the comments of the referees will be reconsidered for publication. Should you disagree with any of the referees' concerns, you should address this in your point-by-point response and provide solid scientific reasons for why you will not make the requested changes.

You should also pay close attention to the editorial comments included below. **In particular, please edit your figure legends to follow Journal standards as outlined in the editorial comments. Failure to do this will result in delays in the re-review process.**

Please note that submitting a revision of your manuscript does not guarantee eventual acceptance, and that your revision will be re-reviewed by the referee(s) before a decision is rendered.

If the revision of the paper is expected to take more than three months, please inform the editorial office. Revisions taking longer than six months may be assessed by new referees to ensure the relevance and timeliness of the data.

Once again, thank you for submitting your manuscript to European Journal of Immunology and we look forward to receiving your revision.

Yours sincerely,
Nadja Bakocevic

On behalf of Prof. Iain McInnes

Dr. Nadja Bakocevic
Editorial Office
European Journal of Immunology
e-mail: ejied@wiley.com
www.eji-journal.eu

****

Reviewer: 1

Comments to the Author
This manuscript presents data on the presence, phenotype and persistence of Cancer/Testis Ag-specific T cells in the peripheral blood of a cohort of young men treated for testicular cancer at a single UK centre.

The major findings are that a significant proportion (albeit a minority overall – 20/45) of patients have detectable IFN-secreting T cells at the time of diagnosis. These are all MAGE-specific (with no NY-ESO-1-specific cells), and are restricted to MAGE-A3 in patients with NSGTT, and the cells react to well-defined MAGE peptides such as EVDP. There is marked reduction in these MAGE-specific cells following treatment. At presentation, patients with testicular tumours have fewer naïve T cells, with an expanded T

effector/memory population compared to healthy volunteers. However, in the patients with cancer, the naïve pool increases over time following treatment as the effector memory population decreases.

It is impressive that the authors have obtained such longitudinal samples from patients and the experiments are well-controlled. Overall, the results are interesting. There are several questions/comments

Essential change
There should be a table of clinical characteristics (age, stage, treatment etc) for the patients.

For discussion
- Has IHC been undertaken to correlate expression of the specific antigens with the presence of specific T cell populations. In particular, the NSGCTT patients only had MAGE A3-reactive T cells and only in patients with metastatic disease – was there demonstrable MAGE A3 expression in the primary tumours in these patients only?
- The authors state in the discussion that large immune infiltrates are seen in ‘virtually every’ case of seminoma. Has CD8 IHC been performed on the primary tumours? Is there are correlation between density of immune infiltrate and the presence of the peripheral T cell populations?
- I realise that the numbers are small, but does the type of post-orchidectomy therapy (radiotherapy vs chemotherapy (and indeed carboplatin AUC7 vs EP)) have any impact upon the fall in CTAg-specific T cells?

Reviewer: 2

Comments to the Author
This manuscript studies the immune response towards onco-testis antigen in the blood testicular cancer patients. A notable proportion of patients displays reactivity against onco-testis antigens. The proportion of naïve cells is decreased at diagnosis and further after chemotherapy. The significance of the decreased onco testis reactivity after chemotherapy is unclear.
On a whole this study provides some new information. However, the data are not always correctly analyzed or displayed. Specifically,
In figure 1: from the manuscript, it is not clear whether the samples from the Healthy Donors have been frozen and thawed before the assay. This should be tested. If not this would be an important problem. Although currently done in the field, the background should not be substracted but shown, as it can vary a lot between samples. The positive controls should be shown. The median is meaningless since only some patients display a response. The proportion of responding patients is the only variable to analyze: delete lines 238, 239, 243- 245, 247.

Figure 3: Although often done in the field, after in vitro restimulation for 10 days the quantitative values are meaningless because they very poorly reflect the ex vivo starting frequency of antigen specific T cells. The only statement that can be made is binary: positive or negative. For instance 8/22 is correct on line 302 but the frequencies on line 303 should not be discussed. The same apply to others antigens.

In figure 5, box plots are also widely used but in the opinion of this reviewer, dots for each patients should be used as recommended by Tracey L. Weissgerber , Natasa M. Milic, Stacey J. Winham, Vesna D. Garovic PLoS Biol 13(4): e1002128 DOI: 10.1371/journal.pbio.1002128. Since the proportion of naïve/effector/memory varies a lot according to demographic data, the characteristics of the controls should be provided in a supplementary table.

Other comments:

On page 5 line 78, since onco testis antigen are expressed in the thymus (B Kiewsky data) some level of tolerance is present contrary to what is written.

On line 282: the statement "require involvement" is unsupported by the data: what means "robust immune response" since there is no tumor assessment. The authors may speak that a "coordinated CD4 and CD8 response" seems to be at play but they should not go further.

<u>First revision – authors' response – 17 March 2017</u>

Reviewer 1

Essential change:

1. As requested by Reviewer 1 (and 2) as an essential addition, we have included a table of clinical characteristics which we have included as Supplementary Table. 1. The table includes age, stage, and treatment of patients and demographics of the healthy male control cohort.

For Discussion:

2. As a point for discussion, the Reviewer raises a very interesting point regarding the correlation of MAGE-A antigen expression and the presence of the specific T cell population. Unfortunately, in this study, blood samples with matched tumor tissue were unavailable due to orchidectomy having been performed (outside our institution) prior to patient consent for participation in our research study. In the Discussion section (page 23), we have added a paragraph to address this limitation.

3. Similarly, the lack of matched tumour tissue means we cannot correlate the density of CD8+ T cells with the presence of the peripheral T cell populations. It has however been shown in the literature that a large proportion of infiltrating T cells in TGCTs are activated CD8+ T cells. Importantly, some MAGE-A3 expressing tumours have been shown the comprise MAGE-A3 specific CD8+ T cells (Hadrup SR, 2006).

4. Comparing the impact of different treatment regimes on the observed decline in MAGE-specific T cells would be interesting to investigate in a large cohort of patients. Unfortunately, analysis in this respect is not possible due to the small sample size and the fact that the majority of patients have received the same treatment regime. Specifically, 10 seminoma patients with a response were analyzed (consisting of 18 different responses in total) with 9/10 being stage 1 (carboplatin AUC7) and 1/10 being stage 2b (3 x EP120).

Reviewer 2

Changes to the way data is displayed and analyzed:

1. The major concern of this reviewer was whether the healthy donor IFNγ ELISPOT was performed on fresh or frozen PBMC. Crucially, ELISPOT setup and analysis of peptide stimulated PBMC from healthy donors was identical to that of TGCT patients. Specifically, previously cryopreserved healthy donor and patient PBMC were rested overnight prior to ELISPOT analysis. This was performed to increase the signal-to-noise ratio. We have edited the Materials and Methods section to clarify this point.

2. In the opinion of this reviewer, the 'background should not be subtracted but shown' in the ELISPOT assay. We agree that the background level does vary between samples but DMSO only negative control wells in our cohort all showed fewer than 58 spots / 106 cells. As is frequently done in the field (and in publications in EJI e.g. Winstone, N. 2009; Hopkins, R. 2011; Kelly, C. 2015), we have presented the response minus the background level to demonstrate specifically CTAg reactive cells only, and believe this is far more informative. We have included the CEFT positive control results (Fig. 1F) as requested by the Reviewer.

3. Following the advice from the reviewer (relating to Fig. 3B), statistical analysis of the median response size was removed as only some patients had a response. Lines in the body text of the Results section and in the Figure Legends were removed as recommended.

4. Although it is often done in the field, the absolute frequency of dextramer responses expressed as a percentage of total CD8 T cells have been removed, as recommended by the Reviewer, since these values may not reflect the ex vivo starting frequencies due to short-term in vitro expansion. Our results do

however demonstrate that patients with testicular cancer do generate responses to well-defined MAGE peptides, which have the ability to proliferate and retain effector function in vitro.

5. As we are analysing continuous data from a small sample size, we have now included each patient as a separate scatter-dot within the box and whisker plots as suggested by the Reviewer.

6. As mentioned above we have included a table of clinical characteristics of patients and healthy donors which we have included as Supporting Information Table. 1.

Other Comments:

7. The reviewer highlighted the evidence for expression of CTAgs in the thymus (B Kiewsky, 2004). We understand that expression of MAGE-A family proteins by mTEC could lead to the display of MAGE-A derived peptides to developing thymocytes and consequently result in some level of tolerance. Nevertheless, our data clearly demonstrates the existence of MAGE specific T-cells in peripheral circulation by direct pMHC-I binding and specific secretion of inflammatory cytokines following peptide stimulation, suggesting ‘incomplete’ clonal deletion of this class of antigens. In line with the Reviewers comment, we have modified the referred paragraph to highlight that central tolerance to CTAgs is ‘incomplete’ and not absent as stated previously.

8. Finally, as per the Reviewers recommendation, the statement on page 14 (end of first paragraph) describing the relationship between MAGE-specific CD4 and CD8 T-cell responses was changed to ‘Nonetheless, a strong positive relationship within individual donors was observed between the frequency of MAGE-specific CD4+ and CD8+ T-cells (r=0.7697, p=0.0156) (Fig. 2C) suggesting the development of a coordinated CD4+ and CD8+ T-cell response against such antigens.’ to more accurately summarize these data.

<u>Second Editorial Decision – 13 April 2017</u>

Dear Dr. Pearce,

It is a pleasure to provisionally accept your manuscript entitled "Spontaneous CD4⁺ and CD8⁺ T cell responses directed against cancer testis antigens are present in the peripheral blood of testicular cancer patients" for publication in the European Journal of Immunology. For final acceptance, please follow the instructions below and return the requested items as soon as possible as we cannot process your manuscript further until all items listed below are dealt with.

Peer review correspondence

Please note that EJI articles are now published online a few days after final acceptance (see Accepted Articles: http://onlinelibrary.wiley.com/journal/10.1002/(ISSN)1521-4141/accepted). The files used for the Accepted Articles are the final files and information supplied by you in Manuscript Central. You should therefore check that all the information (including author names) is correct as changes will NOT be permitted until the proofs stage.

We look forward to hearing from you and thank you for submitting your manuscript to the European Journal of Immunology.

Yours sincerely,
Eloho Etemire

on behalf of Prof. Iain McInnes

Dr. Eloho Etemire
Editorial Office
European Journal of Immunology
e-mail: ejied@wiley.com
www.eji-journal.eu
